# A systematic review and meta-analysis of acupuncture versus sham/placebo acupuncture for postoperative gastrointestinal dysfunction in cancer patients: Evidence from randomized controlled trials

**DOI:** 10.1097/MD.0000000000047305

**Published:** 2026-01-23

**Authors:** Mohao Zhu, Lin Chen, Xiaolong Peng, Mengxue Liu, Ying Liu, Weiai Liu

**Affiliations:** a The Second Affiliated Hospital of Hunan University of Chinese Medicine, Changsha, Hunan, China; b The Second Clinical College of Hunan University of Chinese Medicine, Changsha, Hunan, China; c The Second Xiangya Hospital of Central South University, Changsha, Hunan, China.

**Keywords:** acupuncture, cancer, meta-analysis, postoperative gastrointestinal dysfunction, randomized controlled trials, sham/placebo acupuncture

## Abstract

**Background:**

Acupuncture has been found to be an effective treatment for postoperative gastrointestinal dysfunction (PGD). Nonetheless, it remains uncertain if acupuncture possesses a placebo effect. This study compared the efficacy and safety of acupuncture against sham/placebo acupuncture for PGD in cancer.

**Methods:**

We comprehensively searched the Central Register of Controlled Trials of 8 databases from database inception through August 31, 2024 for randomized controlled trials that compared acupuncture therapy with sham/placebo acupuncture. Cochrane risk of bias tool (version 2 of the Cochrane risk of bias tool [RoB2]) was used to analyze risk of bias. Data analysis was performed with Review Manager 5.4, Stata 12.0 software was used to test the publication bias.

**Results:**

Eleven randomized controlled trials involving 1923 patients were included in this study. Meta-analysis results showed that acupuncture therapy was superior to sham/placebo acupuncture in terms of improving time to first flatus (TFF), time to first defecation (TFD), time to bowel sound recovery, length of hospital stay (LOS). The subgroup analysis based on the type of acupuncture and sham acupuncture showed that transcutaneous electrical acupoint stimulation could significantly reduce the TFF, LOS, TFD, and time to bowel sound recovery; electroacupuncture could significantly reduce the TFF and TFD, and there was no significant statistical difference between electroacupuncture and sham acupuncture in LOS. No trial reported severe adverse events of acupuncture.

**Conclusions:**

The results of this study indicate that acupuncture shown greater efficacy than sham/placebo acupuncture in the treatment of PGD in cancer. Acupuncture appeared safe, but adverse events were underreported.

## 1. Introduction

Cancer is among the most lethal diseases affecting humans, and is the 2nd cause of death throughout the world.^[1]^ The global statistics for the year 2022 indicate that there were almost 20 million new cases of cancer and close to 10 million cancer deaths. Demographics-based predictions indicate that the annual number of new cases of cancer will reach 35 million by 2050, a 77% increase from the 2022 level.^[2]^ Cancer seriously affects human health and brings a huge burden to families and society.

Surgery, chemotherapy, and radiotherapy are the principal clinical treatment modalities for cancer. Surgery, being the primary therapy method, greatly enhances disease management and prolongs patient survival.^[3]^ The postoperative period frequently presents several complications, such as insomnia, pain, gastrointestinal dysfunction, and anastomotic fistula.^[4,5]^ Among them, postoperative gastrointestinal dysfunction (PGD) is one of the most common postoperative complications.^[6]^ PGD is characterized by an interruption of bowel motility, which prevents effective intestinal transit and tolerance of food intake, which leads to varying degrees of impaired gastrointestinal function in patients. PGD mainly manifests as nausea, vomiting, abdominal distension, delayed farting, delayed defecation, intestinal obstruction, and gastrointestinal bleeding. Patients with PGD experience higher average social costs, including medical expenses, compared to those without PGD. It aggravates the economic burden of patients, prolongs hospital stays, seriously affects their quality of life, and even increases postoperative morbidity and mortality.^[7]^ Considering the hazards and adverse effects of PGD, it is essential to maximize the improvement of PGD symptoms to promote postoperative recovery and improve postoperative survival.

Acupuncture is a traditional Chinese medical treatment that alleviates gastrointestinal symptoms by activating certain acupoints. Acupuncture is extensively employed in therapeutic settings for a range of gastrointestinal disorders, including constipation, diarrhea, stomach pain, and dyspepsia.^[8,9]^ Multiple meta-analyses indicate that acupuncture-related therapies, including electroacupuncture (EA) and transcutaneous electrical acupoint stimulation (TEAS), effectively enhance gastrointestinal function.^[10,11]^ Nonetheless, physical therapies are particularly prone to inducing placebo effects in clinical trials due to their inherent nature and procedural characteristics, such as direct patient interaction or contextual factors within the therapeutic environment.^[12]^ Acupuncture, as a physical intervention, inevitably exhibits a placebo effect in certain clinical investigations.^[13]^ Sham acupuncture, also termed placebo acupuncture, is defined in the WHO Guidelines for Clinical Research on Acupuncture as a control method simulating acupuncture needle insertion without actual penetration, ideally inducing no physiological response. It mandates that sham/placebo acupuncture controls must be indistinguishable from real acupuncture and elicit no therapeutic effects.^[14]^ While certain meta-analyses indicate that acupuncture effectively enhances time to first flatus (TFF) and time to first defecation (TFD) in PGD,^[15,16]^ there is a lack of systematic reviews comparing the efficacy of acupuncture versus sham/placebo acupuncture in treating PGD.

The aim of this systematic review was to evaluate the efficacy and safety of acupuncture versus sham/placebo acupuncture in treating PGD, thereby offering evidence for clinical recommendations regarding acupuncture in this context.

## 2. Methods

This study followed the guidelines of the Preferred Reporting Items for Systematic Reviews and Meta-Analysis (PRISMA 2020) statement^[17]^ and the protocol has been registered with PROSPERO (Registration number: CRD42024585835).

### 2.1. Search strategy

Two authors (MZ and LC) independently performed a systematic literature search of the following databases: PubMed, Web of Science, Cochrane Library, Embase, China National Knowledge Infrastructure, Chinese Biomedical Literature Database, WanFang Database, and Chongqing VIP Database.

The search strategy comprised 3 components, which were as follows: clinical condition; intervention; study type. The literature search was limited to journal articles published from inception to August 31, 2024. The PubMed search strategy is presented in Table 1. Detailed search strategies can be found in File S1, Supplemental Digital Content, https://links.lww.com/MD/R197.

**Table 1:** Search strategy for the PubMed database.

No.	Search items
#1	Neoplasms [MeSH Terms]
#2	((((((((((((Tumor [Title/Abstract]) OR (Neoplasm [Title/Abstract])) OR (Tumors [Title/Abstract])) OR (Neoplasia [Title/Abstract])) OR (Neoplasias [Title/Abstract])) OR (Cancer [Title/Abstract])) OR (Cancers [Title/Abstract])) OR (Malignant Neoplasm [Title/Abstract])) OR (Malignancy [Title/Abstract])) OR (Malignancies[Title/Abstract])) OR (Malignant Neoplasms [Title/Abstract])) OR (neoplasm malignant [Title/Abstract])) OR (neoplasms malignant [Title/Abstract])
#3	**#1 OR #2**
#4	Postoperative Complications [MeSH Terms]
#5	(((((Postoperative [Title/Abstract])) OR (Postoperative [Title/Abstract])) OR (Post-surgical [Title/Abstract])))
#6	**#4 OR #5**
#7	Gastrointestinal Diseases [MeSH Terms]
#8	(((((Gastrointestinal Disease [Title/Abstract])) OR (Gastrointestinal Disorders [Title/Abstract])) OR (Gastrointestinal Disorder [Title/Abstract])) OR (Gastrointestinal Dysfunction [Title/Abstract]))
#9	**#7 OR #8**
#10	Acupuncture Therapy [MeSH Terms]
#11	((Acupuncture Treatment [Title/Abstract]) OR (Pharmacoacupuncture Treatment [Title/Abstract])) OR (Pharmacoacupuncture Therapy [Title/Abstract])
#12	**#10 OR #11**
#13	(((((((((((Acupuncture [Title/Abstract]) OR (Electroacupuncture [Title/Abstract])) OR (Electroacupuncture [Title/Abstract])) OR (Electric acupuncture [Title/Abstract])) OR (Acupoint [Title/Abstract])) OR (Ear Needle [Title/Abstract])) OR (Auricular Needle [Title/Abstract])) OR (Wrist Ankle Needle [Title/Abstract])) OR (Acupoint Embedding [Title/Abstract])) OR (Acupoint Injection [Title/Abstract])) OR (Acupoint Sticking [Title/Abstract])) OR (Needle Knife [Title/Abstract])
#14	**#12 OR #13**
#15	Randomized Controlled Trial [MeSH Terms]
#16	((((Clinical Study [Title/Abstract])) OR (Clinical Trial [Title/Abstract])) OR (Controlled Clinical Trial [Title/Abstract]))
#17	placebo [Title/Abstract]
#18	**#15 OR #16 OR #17**
#19	**#3 AND #6 AND #9 AND #14 AND #18**

### 2.2. Inclusion and exclusion criteria

Participants: the study included patients diagnosed with cancer (any type) and underwent surgery (any type of surgery, including open or laparoscopic surgery). Participants aged 18 years or older will be included in the study. Patients with gastrointestinal dysfunction such as dyspepsia, gastritis, ulcerative diseases, and others were excluded.

Intervention (s): the interventions included various forms of acupuncture therapies, such as EA (connecting electrodes to acupuncture needles after the needles have stimulated acupoints, and generating weak electrical stimulation), transcutaneous electrical stimulation on acupoints (TEAS, using surface electrodes on skin acupoints, delivering low-frequency weak electrical currents to stimulate the acupoints and underlying tissues), etc. Or acupuncture therapies combined with routine treatment. Routine treatment included usual postoperative care, fasting, gastrointestinal decompression, anti-infection, electrolyte acid–base imbalance correction, and nutritional support, etc. Studies that do not contain acupuncture or acupuncture combined with other therapies, or received traditional Chinese medicine (TCM) were excluded in this review.

Comparator: the control groups in the studies included sham acupuncture or placebos acupuncture. Studies in which do not contain sham acupuncture or placebos acupuncture in the control groups were excluded.

Outcomes: outcome measures included at least one of the following: the primary outcomes of this study were TFF and TFD. The secondary outcomes were time to bowel sound recovery (TBSR), length of hospital stay (LOS) and adverse events of acupuncture treatment (such as nerve injury, and extreme fainting).

Study type (s): only randomized controlled trials (RCTs) were eligible for inclusion in this review.

Non-English articles, studies with repeated data or secondary analysis, studies from non-RCTs (including animal studies, master’s and doctoral dissertations, books, protocols, conference abstracts, case reports, correspondences, overviews, or systematic reviews), and those studies whose outcome indicators did not match were not considered.

### 2.3. Data extraction

Screening of data was performed using Endnote X9. Studies with duplicate titles were deleted by both Endnote and manual screening. Two investigators (MZ and LC) independently extracted data from the included studies. The extracted data included the 1st author’s name, publication year, cancer type, sample size, patient’s age, sex ratio, type of intervention, type of control intervention, intervention duration, acupuncture points, main outcomes, adverse events. The summary of included studies is shown in Table 2.

**Table 2:** Summary of included studies.

Study	Cancer type	Sample size (I vs C)	Age (yr)	Sex ratio (M: F)	Intervention group therapy	Control group therapy	Duration and frequency of treatment	Acupuncture points	Outcomes
Wang et al^[20]^	Colorectal cancer	248 (125 vs 123)	60.2 ± 11.0 vs 60.2 ± 11.8	79:46 vs 83:40	EA	Sham EA with non-therapeutic acupoints	From postoperative day 1 to day 4, 30 min, once daily	ST36, ST37, RN12, ST25	TFF, TFD, LOS
Li et al^[21]^	Colorectal cancer	96 (48 vs 47)	58.12 ± 7.34 vs 56.45 ± 7.26	25:23 vs 29:18	TEAS	Sham TEAS with same acupuncture acupoints	30 min before anesthesia induction to the end of surgery	ST36, LI4, SP6, PC6	TFF, TFD, LOS
Li et al^[22]^	Abdominal tumor	306 (154 vs 152)	60 ± 13 vs 60 ± 12	85:69 vs 96:56	TEAS	Sham TEAS with non-therapeutic acupoints	30 min before surgery until the end of the operation, and from postoperative day 1 to day 4, 30 min, once daily	LI4, PC6, ST36, ST37	TFF, LOS, TBSR
Gao et al^[23]^	Colorectal cancer	610 (303 vs 307)	61 ± 11.9 vs 62.3 ± 11.9	176:127 vs 156:151	TEAS	Sham TEAS with same acupuncture acupoints	From postoperative day 1 to day 3, 30 min, once daily	ST36, ST37, SP6	TFF, TFD, TBSR
Ding et al^[24]^	Gastric cancer	60 (30 vs 30)	54.23 ± 7.98 vs 55.57 ± 10.63	18:12 vs 17:13	EA	Sham EA with non-therapeutic acupoints	30 min preoperative, and from postoperative day 1 to day 5, 30 min once daily	ST36, ST25, DU20, PC6	TFF, TFD, LOS
Zhu et al^[25]^	Liver cancer	74 (37 vs 37)	63.4 ± 1.7 vs 63.4 ± 1.5	31:6 vs 25:12	TEAS	Same TEAS stimulation parameters with non-therapeutic acupoints	From postoperative day 1 to day 3, 1 h, twice daily	ST36, PC6	TFD
Zhang et al^[26]^	Rectal cancer	90 (45 vs 45)	64 ± 12 vs 65 ± 11	28:17 vs 31:16	TEAS	Sham TEAS with same acupuncture acupoints	After operation and from postoperative day 1 to day 3, 30 min, once daily	ST36, ST37, SP6	TFF, LOS, TBSR
Song et al^[27]^	Rectal cancer	92 (46 vs 46)	61.5 ± 7.2 vs 60.1 ± 6.8	31:15 vs 28:17	TEAS	Sham TEAS with same acupuncture acupoints	30 minutes before anesthesia induction to the end of surgery	DU20, ST36, SP6, PC6	TFF, TFD, TBSR
Li et al^[28]^	Cervical carcinoma	120 (60 vs 60)	53.4 ± 11.6 vs 52.8 ± 13.7	0:60 vs 0:60	TEAS	Sham TEAS with same acupuncture acupoints	After surgery and from postoperative day 1 to day 3, 30 min, once daily	DU20, PC6, SP6	TFF, TFD, LOS
Gu et al^[29]^	Gastric cancer	117 (59 vs 58)	56.67 ± 6.23 vs 57.59 ± 7.32	29:30 vs 31:27	TEAS	Sham TEAS with same acupuncture acupoints	30 min before anesthetic induction to 30 min after the operation, and from day 1 to day 2, 3 times daily	ST36, PC6	TFF, TFD, TBSR
Ng et al^[30]^	Colorectal cancer	110 (55 vs 55)	67.4 ± 9.7 vs 67.4 ± 10.7	35:20 vs 33:22	EA	Sham EA with non-therapeutic acupoints	From postoperative day 1 to day 4, 20 min, once daily	ST36, LI4, SP6, PC6	TFF, TFD, LOS

C = control group; EA = electroacupuncture; F = female; I = intervention group; LOS = length of hospital stay; M = male; TBSR = time to bowel sound recovery; TEAS = transcutaneous electrical acupoint stimulation; TFF = time to first flatus; TFD = time to first defecation.

**Figure 1.**
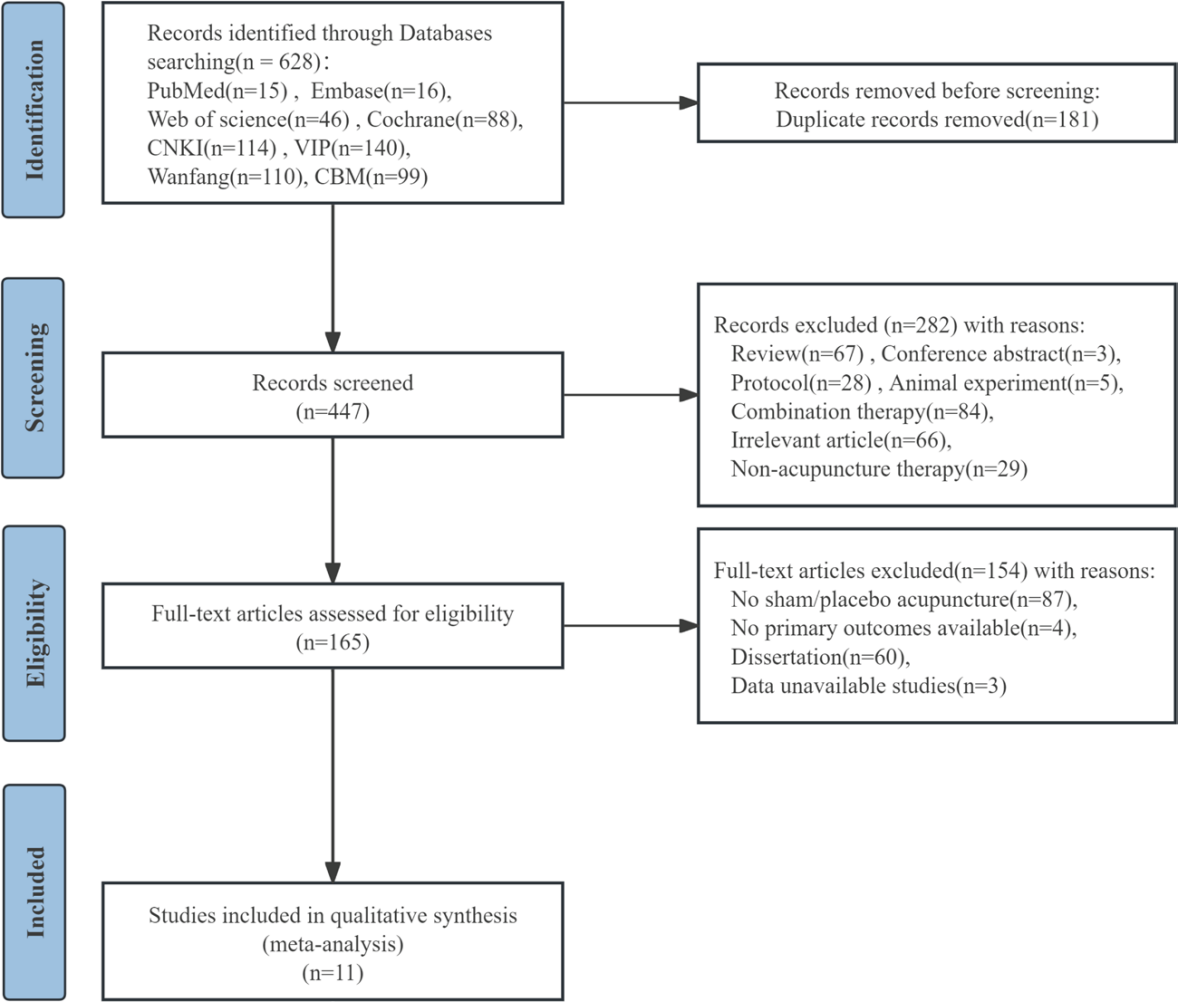
The PRISMA flowchart describing the selection process. PRISMA = Preferred Reporting Items for Systematic Reviews and Meta-Analysis.

Two researchers (MZ and LC) independently reviewed the titles and abstracts using the inclusion and exclusion criteria for initial inclusion. Disagreements between the 2 researchers were resolved by further review of the full text and discussion with a third researcher (WL). The screening and selection process is detailed in a PRISMA flow chart shown in Figure 1.

### 2.4. Risk of bias assessment

To evaluate the risk of bias in the selected studies, 2 independent reviewers (MZ and LC) applied version 2 of the Cochrane Risk of Bias tool (version 2 of the Cochrane risk of bias tool [RoB2]).^[18]^ The evaluation covered 6 domains: randomization process; deviations from intended interventions; missing outcome data; outcome measurement; selection of reported results; and overall bias. Each domain was classified as low, high, or of some concern for bias. Disagreements were discussed between the 2 reviewers, and if these were unresolved, a 3rd reviewer (WL) participated in the discussion until a consensus was reached.

### 2.5. Data analysis

The meta-analysis was performed using Review Manager (RevMan) software, Version 5.4 (The Cochrane Collaboration, London, United Kingdom) by 2 authors (MZ and ML). Any disagreements between the 2 authors were resolved through discussion with the third experienced author of the review (WL). Continuous variables were assessed using the mean difference (MD) with a 95% confidence interval (CI) when the unit was the same. For outcomes with differing units, the standardized MD were employed. Statistical significance was set at *P* < .05. The magnitude of the effect size of the standardized MD was rated as follows: ≤0.2 indicated a small effect, 0.5 indicated a moderate effect, and ≥0.8 indicated a large effect. Forest plots were used to assess heterogeneity using the *I*^2^ statistic, with *I*^2^ ≥50% indicating significant heterogeneity. *I*^2^ <50% was considered indicative of low heterogeneity, and a fixed-effect model was employed. *I*^2^ ≥50% was considered indicative of high heterogeneity, and a random-effects model was employed.

If a significant heterogeneity existed between a set of studies, causes of heterogeneity, such as patient characteristics and the degree of variation in the interventions, were explored. The primary outcomes were analyzed through subgroup analysis. Sensitivity analysis was performed by excluding each RCT sequentially and comparing the model characteristics to test the robustness of the result. A funnel plot was used to assess the reporting biases if more than 10 trials were included in the meta-analysis. The asymmetry of the funnel plots was evaluated using Egger tests, and a *P*-value of < .05 represented significant publication bias.^[19]^

### 2.6. The quality of evidence

Two authors (MZ and LC) used the Grading of Recommendations, Assessment, Development, and Evaluation (GRADE) to assess the quality of evidence. The assessment classified the evidence into 4 levels: high, moderate, low, and very low, any disagreements between the 2 authors were resolved through discussion with the third experienced author of the review (WL).

## 3. Results

### 3.1. Search results

A total of 628 potentially relevant articles were identified in the initial database search. After eliminating 181 duplicates, 447 articles were screened, following which 433 articles were eliminated based on the title and abstract screening process. Furthermore, 3 studies were excluded after reviewing full texts based on the eligibility criteria. Eventually, 11 studies^[20–30]^ were included in this meta-analysis (Fig. 1)

### 3.2. Study characteristics

The included RCTs were published between 2013 and 2023. The main characteristics of the included studies are summarized in Table 2.

#### 3.2.1. Patient characteristics

The 11 studies^[20–30]^ comprised 1923 participants, with 962 assigned to the intervention group and 961 to the control group. All participants were diagnosed with cancer, underwent surgery, and subsequently developed PGD. Regarding cancer types, 9 studies^[20–24,26,27,29,30]^ focused on gastrointestinal tumors (gastric and colorectal cancer), 1 study^[25]^ on liver cancer, and 1 study^[28]^ on cervical carcinoma.

#### 3.2.2. Intervention characteristics

Two types of acupuncture techniques were involved: EA,^[20,24,30]^ TEAS.^[21–23,25–29]^ Nine acupoints were used across all studies. The commonly used acupoints were stomach (ST) 36, ST37, ST25, pericardium 6, spleen 6, and large intestine 4, which were mainly in the stomach meridian and spleen meridian. In terms of combining acupoints, 9 studies^[21–23,25–30]^ used a combination of distal acupoints, and 2 studies^[20,24]^ used a combination of distal–proximal acupoints. All acupuncture treatments were performed during the peri-operative period (The continuous period from the decision to undergo surgery, through the surgical procedure itself, to the patient’s basic recovery). Acupuncture retention times in most studies varied from 20 to 30 minutes. The treatment was administered once a day or twice a day.

#### 3.2.3. Control characteristics

Five studies used non-therapeutic acupoints (points that have not been proven to possess therapeutic effects by TCM theory or modern clinical research),^[20,22,24,25,30]^ and 6 studies^[21,23,26–29]^ used the same acupoints as the intervention group. One study used the same treatment parameters as the control group with non-therapeutic acupoints. Ten studies^[20–24,26–30]^ disabled therapeutic devices by severing electrode leads or disconnecting power sources to prevent acupoint electrical stimulation. One study^[25]^ maintained device activation using parameters identical to the therapeutic group but use non-therapeutic acupoints.

#### 3.2.4. Outcomes

All studies used TFF, TFD, TBSR, or LOS to assess the effect of acupuncture.

### 3.3. Risk of bias

Regarding the risk of random sequence generation, 7 studies used random number generation,^[21,22,25,27–30]^ 3 studies^[20,23,24]^ used the randomized block design, and 1 study^[26]^ implemented randomization approach but did not detail the specific randomization procedure in the text. Among these, 2^[22,30]^ utilized opaque, sealed envelopes, 1^[20]^ utilized a central randomization system. Due to the distinctive nature of acupuncture interventions, all acupuncturists involved in the therapeutic procedures was not blinded. Among the included studies, 6 studies^[20,23,24,28–30]^ implemented blinding for both study personnel and participants, while the remaining 5 studies^[21,22,25–27]^ lacked description of blinding procedures. Five studies^[21,24,26–28]^ did not explicitly state whether outcome assessors were blinded, suggesting a unclear risk of measurement bias.

Regarding the outcome data, all studies reported complete outcome data, and provided outcome data accompanied by appropriate statistical analyses. Based on this, the trials were assessed as having a low risk of bias.

**Figure 2.**
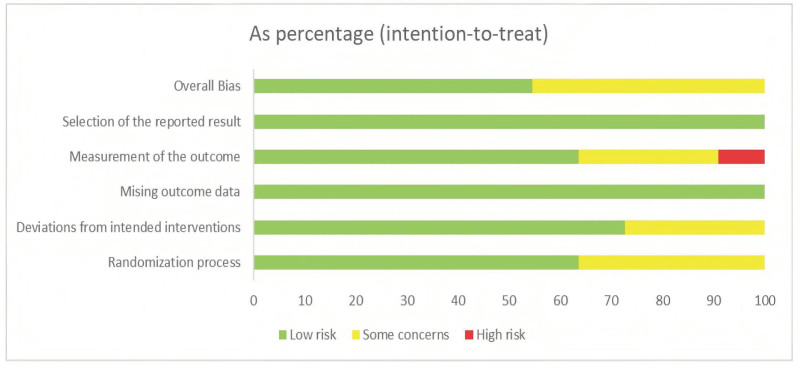
The risk of bias graph.

**Figure 3.**
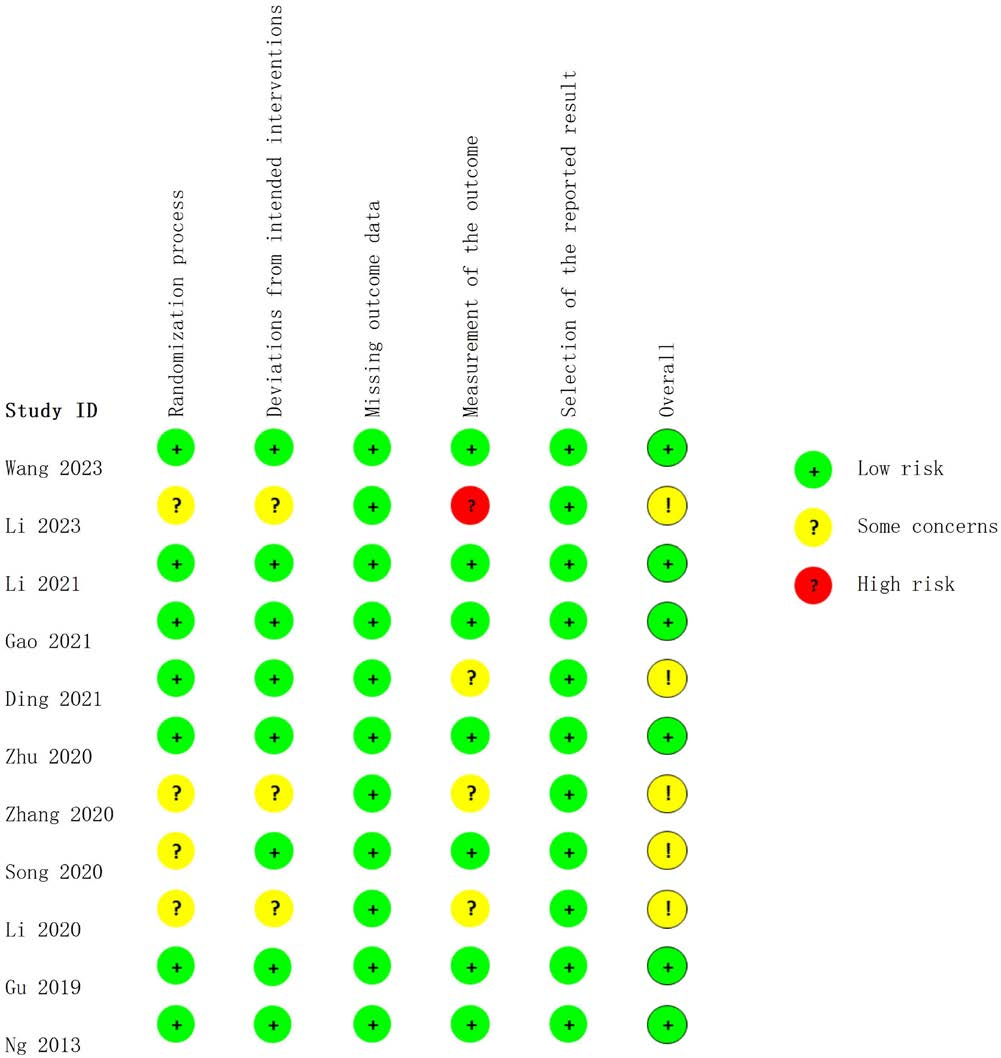
The summary of the risk of bias.

Figure 2 presents the risk of bias in each of the 5 domains for all included studies. A summary of the risk of bias in each of the included trials is presented in Figure 3.

### 3.4. Safety

Acupuncture-related adverse events were documented detail in 1 study,^[20]^ including hematoma, sharp pain, and residual needling sensation after needle removal. These treatment-related adverse events were mild and transient. None of the remaining studies^[21–30]^ provided details regarding adverse events. No severe adverse events were reported. The existing evidence indicates that acupuncture treatment is safe for PGD in cancer.

### 3.5. Meta-analysis

#### 3.5.1. TFF

**Figure 4.**
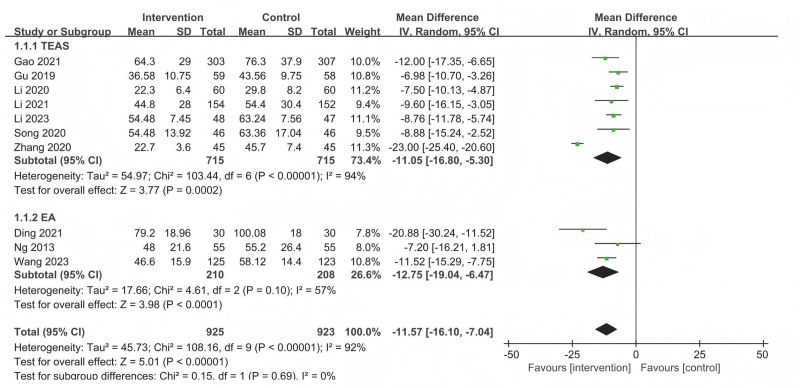
The forest plot of the comparison of acupuncture with sham/placebo acupuncture for TFF. TFF = time to first flatus.

Ten studies used TFF to assess the effect of acupuncture-related therapy on postoperative gastrointestinal function. The pooled results indicated that acupuncture-related therapy had a better effect in shortening the TFF compared with sham/placebo acupuncture (MD = −11.57; 95% confidence interval [CI]: −16.10 to −7.04), but considerable heterogeneity was observed (*P *< .001; *I*^2^ = 90.2%) (Fig. 4).

Subgroup analyses were implemented based on different intervention measures. The results showed that TEAS was superior to sham/placebo acupuncture in improving TFF (MD = −11.5, 95% CI: −16.80 to −5.30), EA (MD = −12.75, 95% CI: −19.04 to −6.47) was superior to sham/placebo acupuncture (Fig. 4).

#### 3.5.2. TFD

**Figure 5.**
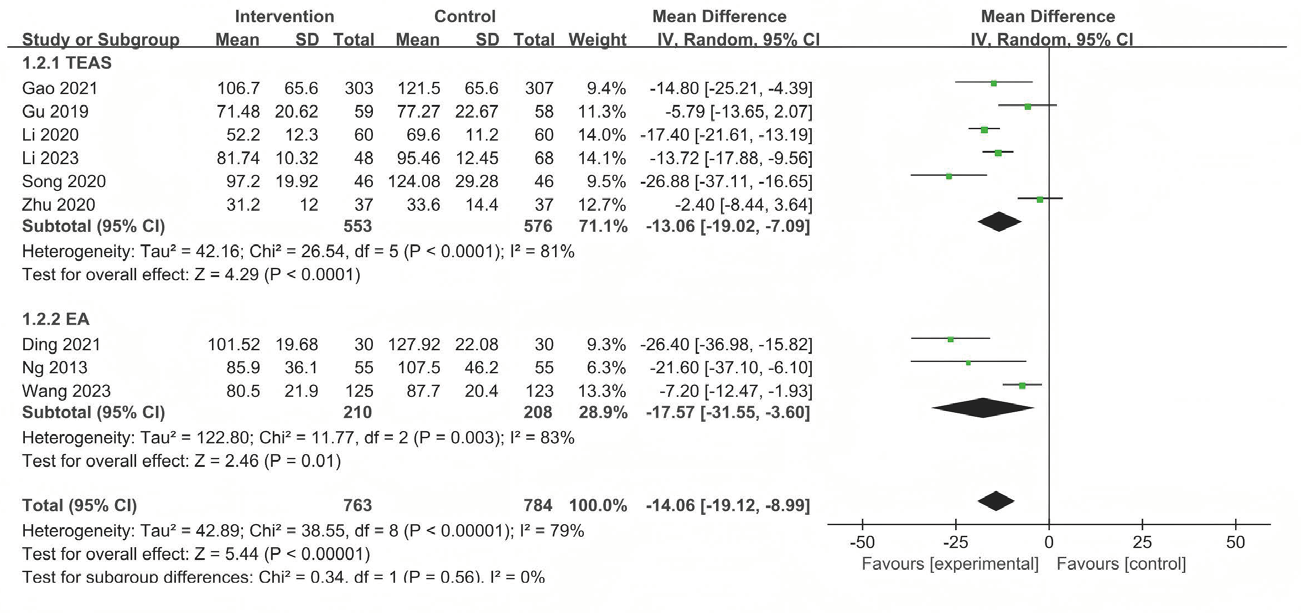
The forest plot of the comparison of acupuncture with sham/placebo acupuncture for TFD. TFD = time to first defecation.

TFD was observed in 9 studies. The analysis revealed that acupuncture-related therapy reduced the TFD compared with sham/placebo acupuncture (MD = −14.06; 95% CI: −19.12 to −8.99), but considerable heterogeneity was observed (*P* < .001; *I*^2^ = 79%) (Fig. 5).

Subgroup analyses were conducted according to intervention modality. TEAS demonstrated superior efficacy versus sham/placebo acupuncture (*P* < .05 for all comparative outcomes (MD = −13.06, 95% CI: −19.02 to −7.09), EA (MD = −17.57, 95% CI: −31.55 to −3.60) was superior to sham/placebo acupuncture (Fig. 5).

#### 3.5.3. LOS

**Figure 6.**
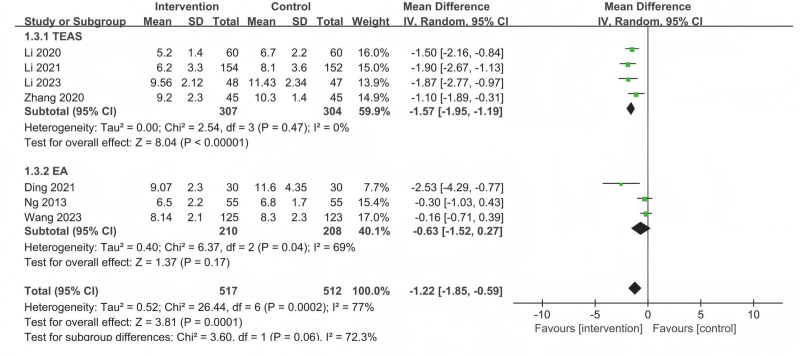
The forest plot of the comparison of acupuncture with sham/placebo acupuncture for LOS. LOS = length of hospital stay.

LOS was observed in 7 studies. The analysis revealed that acupuncture-related therapy reduced the LOS compared with sham/placebo acupuncture (MD = −1.22; 95%CI: −1.85 to −0.59; *P* < .001), but considerable heterogeneity was observed (*P* < .001; *I*^2^ = 77%) (Fig. 6).

Subgroup analyses were implemented based on different intervention measures. The results showed that TEAS was superior to sham/placebo acupuncture (MD = −1.57, 95% CI: −1.95 to −1.19). However, there were no significant statistical difference between EA and sham/placebo acupuncture (MD = −0.63, 95% CI: −1.52 to 0.27) (Fig. 6).

#### 3.5.4. TBSR

**Figure 7.**
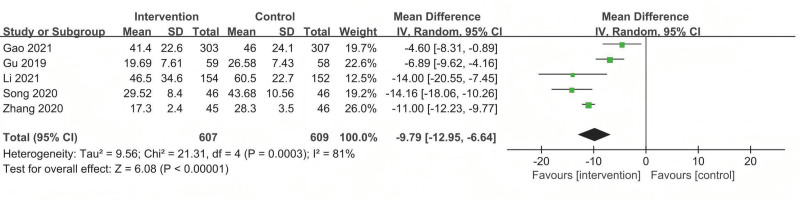
The forest plot of the comparison of acupuncture with sham/placebo acupuncture for TBSR. TBSR = time to bowel sound recovery.

Five studies used TBSR to assess the effect of TEAS therapy on postoperative gastrointestinal function. The pooled results indicated that TEAS therapy had a better effect in shortening the TFF compared with sham/placebo acupuncture (MD = −9.79; 95% CI: −12.95 to −6.64), but considerable heterogeneity was observed (*P* < .001; *I*^2^ = 80.1%) (Fig. 7).

### 3.6. Sensitivity analysis

The leave-one-out method was utilized to perform sensitivity analysis in order to determine the robustness of pooled findings (TFF, TFD, LOS, and TBSR). No changes were observed in the significant outputs from the meta-analysis by omitting a single study in terms of the TFD, LOS, and TBSR. These heterogeneities did not influence the stability of the result. However, after eliminating 1 study,^[26]^ the results related to the TFD (*P* < .001) revealed significance, and the heterogeneity (*I*^2^ = 32%) was significantly reduced (Table 3).

**Table 3:** Sensitivity analysis.

Outcome	Study omitted	Effect estimate	Heterogeneity	
			*P*	*I*^2^ (%)
TFF	Wang et al^[20]^	MD −11.50 [−16.7 to −6.64]	*P* < .001	93
	Li et al^[21]^	MD −11.92 [−17.05 to −6.79]	*P* < .001	92
	Li et al^[22]^	MD −11.78 [−16.64 to −6.91]	*P* < .001	93
	Gao et al^[23]^	MD −11.52 [−16.47 to −6.58]	*P* < .001	93
	Ding et al^[24]^	MD −10.78 [−15.51 to −6.06]	*P* < .001	92
	Zhang et al^[26]^	MD −9.36 [−11.21 to −7.51]	*P* < .001	32
	Song et al^[27]^	MD −11.85 [−16.72 to −6.99]	*P* < .001	93
	Li et al^[28]^	MD −12.08 [−17.06 to −7.10]	*P* < .001	91
	Gu et al^[29]^	MD −12.13 [−17.04 to −7.22]	*P* < .001	92
	Ng et al^[30]^	MD −11.95 [−16.72 to −7.18]	*P* < .001	93
TFD	Wang et al^[20]^	MD −15.15 [−20.73 to −9.57]	*P* < .001	79
	Li et al^[21]^	MD −14.36 [−20.59 to −8.12]	*P* < .001	82
	Gao et al^[23]^	MD −14.04 [−19.54 to −8.54]	*P* < .001	82
	Ding et al^[24]^	MD −12.73 [−17.76 to −7.69]	*P* < .001	78
	Zhu et al^[25]^	MD −15.55 [−20.35 to −10.75]	*P* < .001	72
	Song et al^[27]^	MD −12.62 [−17.60 to −7.64]	*P* < .001	77
	Li et al^[28]^	MD −13.63 [−19.40 to −7.86]	*P* < .001	79
	Gu et al^[29]^	MD −15.13 [−20.55 to −9.71]	*P* < .001	80
	Ng et al^[30]^	MD −13.55 [−18.82 to −8.29]	*P* < .001	81
LOS	Wang et al^[20]^	MD −1.41 [−1.98 to −0.84]	*P* = .02	63
	Li et al^[21]^	MD −1.11 [−1.79 to −0.44]	*P* < .001	78
	Li et al^[22]^	MD −1.09 [−1.76 to −0.43]	*P* < .001	76
	Ding et al^[24]^	MD −1.11 [−1.75 to −0.47]	*P* < .001	79
	Zhang et al^[26]^	MD −1.26 [−2.00 to −0.51]	*P* < .001	81
	Li et al^[28]^	MD −1.18 [−1.91 to −0.45]	*P* < .001	79
	Ng et al^[30]^	MD −1.39 [−2.24 to −0.53]	*P* < .001	81
TBSR	Li et al^[22]^	MD −9.19 [−12.64 to −5.73]	*P* < .001	85
	Gao et al^[23]^	MD −10.95 [−13.94 to −7.96]	*P* < .001	74
	Zhang et al^[26]^	MD −9.54 [−14.14 to −4.94]	*P* < .001	82
	Song et al^[27]^	MD −8.76 [−12.30 to −5.21]	*P* < .001	82
	Gu et al^[29]^	MD −10.65 [−14.41 to −6.89]	*P* < .001	79

### 3.7. Subgroup analysis

Due to the limited number of studies, only the TFF was analyzed. The subgroups were based on the following characteristics: acupuncture techniques: TEAS, EA; cancer types: gastrointestinal tumors (including gastric cancer and colorectal cancer), cervical carcinoma; acupoint combinations: a combination of distal acupoints, a combination of distal–proximal acupoint. As presented in Table 4, subgroup analysis revealed no significant association between the assessed variables and TFF.

**Table 4:** Subgroup analysis.

Outcome	Subgroup		Studies	Patients	Effect estimate	Heterogeneity	
						*P*	*I*^2^ (%)
TFF	Acupuncture therapy	Transcutaneous electrical acupoint stimulation	6	1430	MD −11.05 [−16.80 to −5.30]	*P* < .001	94
		Electroacupuncture	3	418	MD −12.75 [−19.04 to −6.47]	*P* = .10	57
	Cancer types	Gastrointestinal tumors	9	1728	MD −12.08 [−19.04 to −6.47]	*P* < .001	91
		Cervical carcinoma	1	120	MD −7.50 [−10.13 to −4.87]	*P* < .001	-
	Acupoints combination	Combination of distal acupoints	8	1540	MD −10.66 [−16.04 to −5.28]	*P* < .001	93
		Combination of distal-proximal acupoints	2	308	MD −15.18 [−24.13 to −6.23]	*P* = .07	70

### 3.8. Publication bias

**Figure 8.**
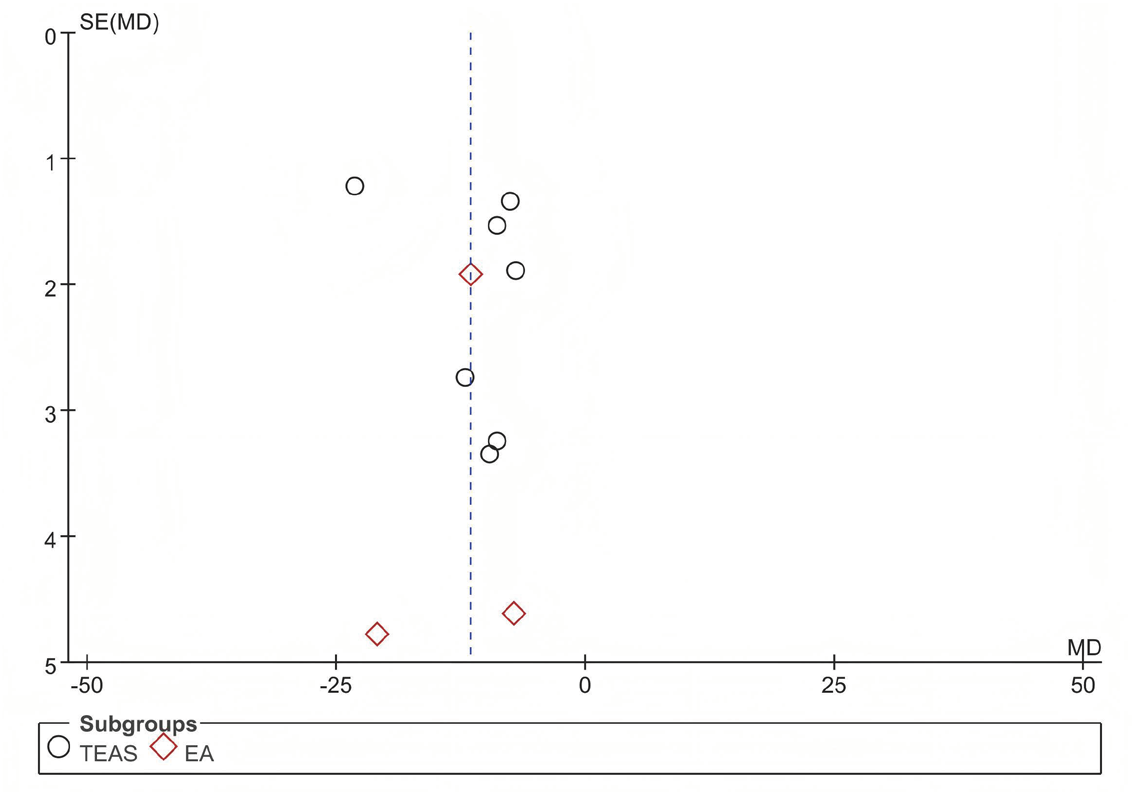
The funnel plot of the comparison of acupuncture with sham/placebo acupuncture for TFF. TFF = time to first flatus.

**Figure 9.**
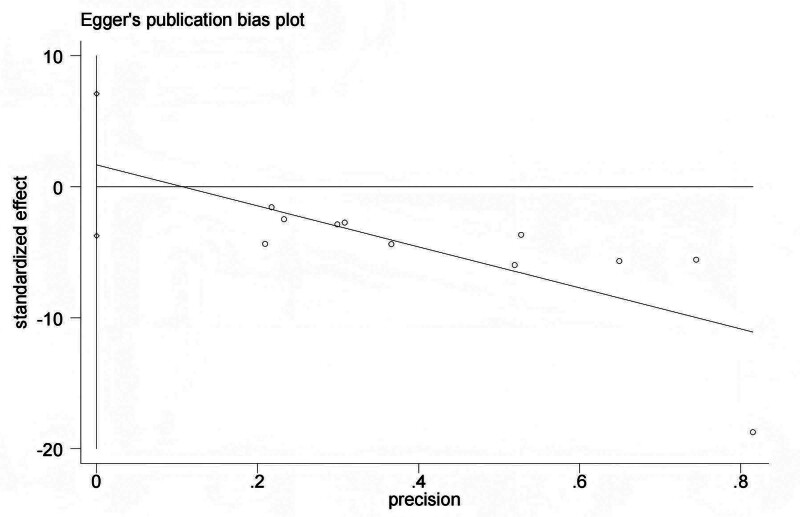
The Egger publication bias plot of the comparison of acupuncture with sham/placebo acupuncture for TFF. TFF = time to first flatus.

The funnel plot of 10 trials included in the meta-analysis for TFF revealed approximate symmetry between them (Fig. 8). Egger test demonstrated no obvious publication bias (TFF: *P* = .503) (Fig. 9).

### 3.9. Quality of evidence

**Figure 10.**
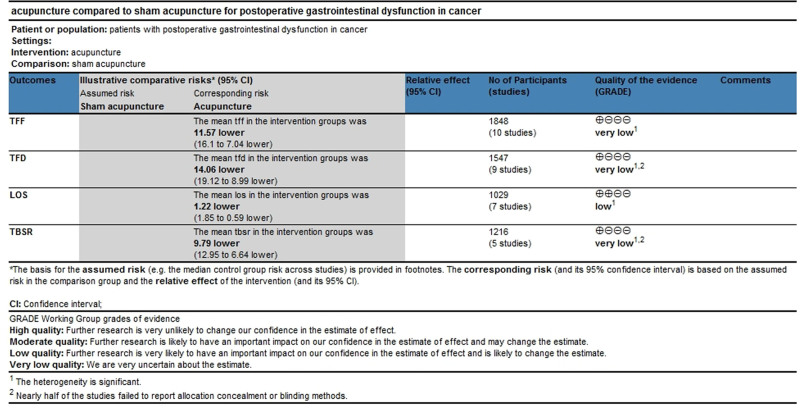
The GRADE Evidence Profile for acupuncture-related therapy versus sham/placebo acupuncture. GRADE = Grading of Recommendations, Assessment, Development, and Evaluation.

A systematic review was conducted using the GRADE approach developed by the Cochrane Collaboration. The systematic analysis included 4 outcomes in the acupuncture therapy group versus the sham/placebo acupuncture group (Fig. 10). The GRADE Evidence Profile indicated that the quality of evidence was low, mainly due to the risk of bias and unexplained inconsistencies.

## 4. Discussion

### 4.1. Summary of main evidence

This systematic review and meta-analysis included 11 RCTs comprising 1923 patients. The results showed that acupuncture was better than sham/placebo acupuncture in improving TFF, TFD, LOS, and TBSR. Transcutaneous electrical acupoint stimulation was superior to sham/placebo acupuncture in TFF, TFD, LOS, and TBSR. Electroacupuncture was better than sham/placebo acupuncture in TFF, TFD, and TBSR. There was no significant statistical difference between EA and sham/placebo acupuncture in LOS. Acupuncture effectively reduced the TFF in all cancer types included in this review. Besides, acupuncture treatment with both a combination of distal acupoints and distal-proximal acupoints could reduce the TFF. The sensitivity analysis and publication bias supported the stability of the overall results. No severe adverse events were reported in any of the trials. In cancer patients with PGD, acupuncture demonstrates significantly greater efficacy compared to sham/placebo acupuncture. Based on comprehensive outcomes of TFF, TFD, LOS, TBSR, and safety assessments, acupuncture demonstrates superior efficacy over sham/placebo acupuncture for PGD.

With the deepening understanding of PGD, recent systematic reviews and meta-analyses have highlighted the benefits of acupuncture for PGD.^[15,16]^ However, these studies included control groups involving sham/placebo acupuncture, pharmacological interventions, or combined acupuncture–drug therapies. No research has specifically compared acupuncture versus sham/placebo acupuncture alone. The innovative aspect of this study lies in its specific comparison of acupuncture versus sham/placebo acupuncture to investigate the therapeutic specificity of acupuncture for PGD, thereby excluding placebo effects. Owing to considerable heterogeneity in acupuncture’s effects on TFF, TFD, LOS, and TBSR, subgroup analysis was performed based on types of acupuncture, which involved 2 types of acupuncture (TEAS, EA).

### 4.2. Possible explanations for the mechanisms of acupuncture

The etiology of PGD is thought to involve a combination of factors, including abnormal sympathetic input, dysregulated hormone release, inflammatory processes, and analgesic effects.^[31]^ Acupuncture, a TCM therapy, has been employed for treating gastrointestinal disorders since ancient times.^[32]^ Its therapeutic mechanism involves stimulating specific acupoints to regulate visceral function through the meridian system, thereby achieving disease-modifying effects.^[33]^ With increasing global clinical and animal research validating its efficacy,^[34,35]^ acupuncture has gained wide acceptance in recent years. Clinical evidence confirms that acupuncture and moxibustion therapies effectively improve intestinal motility, expedite postoperative exhaust and defecation (evidenced by reduced time to 1st flatus and defecation), and shorten hospital stays.^[36–39]^ One study^[40]^ demonstrates that EA activates the vagus nerve through stimulation of the dorsal motor nucleus, thereby initiating the α7nAChR-mediated JAK2/STAT3 signaling pathway in macrophages. This cascade suppresses pro-inflammatory cytokine expression and restores gastrointestinal motility in murine models of postoperative ileus. Hu^[41]^ established that EA modulates jejunal function primarily through parasympathetic pathway activation, with Aδ-fibers and C-fibers constituting pivotal neural mediators in this mechanism. Moreover, EA could modulate the gut microbiota and enhances butyric acid production, thereby promoting intestinal motility.^[42]^ This meta-analysis demonstrates that acupuncture significantly improves TFF, TFD, and TBSR with superior efficacy compared to the sham/placebo acupuncture group, suggesting its potential to enhance gastrointestinal motility.

The mechanism of acupuncture is still under investigation, with potential effects including the enhancement of gastrointestinal peristalsis through various pathways, such as the release of anti-inflammatory substances, regulation of autonomic nervous system activity, and improvement of hormonal metabolism.^[43]^

### 4.3. Problem of sham/placebo acupuncture control and placebo effect

In this systematic review and meta-analysis, all studies were sham/placebo acupuncture in the control group. Placebo control is a commonly used approach in randomized controlled trials, utilized to assess the specific efficacy of interventions. Unlike drug interventions, acupuncture, as a non-pharmacological approach, presents challenges in establishing a qualified placebo control due to its inherent complexity and operational factors.^[44]^ The advancement of acupuncture clinical research has led to the emergence of various forms of acupuncture placebo control. These methodologies are collectively designated as sham acupuncture or placebo acupuncture in clinical trials. At present, the main methods for implementing sham acupuncture and placebo acupuncture include: stimulating non-therapeutic acupoints; applying superficial stimulation to therapeutic or non-therapeutic acupoints; applying blunt pressure to the skin surface; using nonirritating comfort devices (a specific device was attached to the skin, and the needles only pierced the device without producing any stimulation). In this study, sham/placebo acupuncture specifically refers to methods 1 and 4 above.

The placebo response to treatments is increasingly recognized in clinical interventions, including all health alterations resulting from the administration of inert interventions, as well as the natural progression of the disease.^[45]^ Placebo effects are associated with multifaceted elements like doctor-patient interaction, clinical setting, individual differences, and cognitive processes, representing a synthesis of physiological and psychological effects.^[46]^ The placebo effect of acupuncture therapy was recognized in initial tests, demonstrating a distinct placebo effect in some diseases.^[47,48]^ In this study, the subgroup analysis results indicated no significant difference between EA and sham/placebo acupuncture in enhancing LOS, implying that EA may exhibit placebo effects on LOS improvement. The placebo effect may be associated with the type of sham/placebo acupuncture. Research shows that different forms of sham/placebo acupuncture vary in effect size, with skin-penetrating sham acupuncture demonstrating greater effects than non-penetrating approaches.^[49]^ Both skin-penetrating and non-penetrating sham acupuncture interventions involve localized skin contact, thereby activating the acupoint tissue microenvironment. This results in physiological signal transduction and partial neurobiological effects analogous to those elicited by real acupuncture, making it challenging to eliminate the specific therapeutic effects of placebo acupuncture. Consequently, designing a placebo needle that fully excludes acupuncture-specific effects remains difficult in clinical trials.^[13,50]^ Two of the sham/placebo acupuncture controls included in this meta-analysis employed superficial needling via the dermis, which may explain its placebo effects.

Some researchers have indicated that the placebo effect is contingent upon various outcomes. The results of a randomized controlled trial on asthma indicated that sham/placebo acupuncture exhibited no significant placebo effect on objective measures; however, there was no notable difference in subjective outcomes between the sham/placebo acupuncture and intervention groups.^[51]^ LOS is susceptible to the subjective assessment of symptoms by physicians or patients, potentially influencing the placebo effect. Consequently, choosing suitable sham/placebo acupuncture modalities and criteria based on the specific objectives of clinical trials may be an effective strategy to mitigate the biased influence of the placebo effect.

### 4.4. Limitations and implications for future research

Although this meta-analysis demonstrated a statistically significant advantage of acupuncture over sham/placebo acupuncture, yet, several limitations warrant consideration. First, substantial heterogeneity was observed. Despite conducting subgroup and sensitivity analyses, the sources of heterogeneity (potentially attributable to variations in acupuncture modalities, cancer types, sample sizes, the number of included studies, and other factors) remain incompletely elucidated. Second, the evidence quality was low. Furthermore, the limited number of trials and small sample size may collectively compromise the robustness of our conclusions. High-level evidence regarding placebo effects in acupuncture trials remains scarce. Third, all included studies were conducted in Chinese populations. Caution is required when extrapolating these findings to other ethnic groups, particularly given potential differences in pharmacogenomic profiles and treatment responsiveness. Therefore, it is necessary to conduct prospective cohort studies and basic research to obtain more conclusive evidence

Acupuncture therapy show significant variations in acupoint selection, stimulation intensity, frequency, and treatment duration, potentially introducing bias. For instance, despite strict standardization of acupoint selection and manipulation methods in trial protocols, deviations in needling depth and stimulation intensity may still occur during implementation due to variations in practitioners’ experience and technique. Meanwhile, in clinical acupuncture practice, practitioners frequently tailor acupoint combinations and therapeutic techniques based on individual patient conditions. In contrast, RCTs often employ fixed protocols with predetermined acupoints and procedures. Such non-individualized approaches, which diverge from real-world clinical practice, may compromise therapeutic outcomes. Consequently, the bias may influence our results.

High-level evidence regarding the impact of the placebo effect on the efficacy of acupuncture therapy remains insufficient. Multi-center, large-sample, high-quality randomized controlled trials are necessary to validate acupuncture therapy’s effectiveness. Future clinical research should standardize the acupuncture control scheme to mitigate the placebo effect. This includes giving a comprehensive and detailed description of the sham/placebo acupuncture control scheme (report the rationale for sham/placebo acupuncture design, strictly control stimulation sites and intervention methods, and apply specific needle devices) a blank or waiting group for efficacy comparison, and establish diversified indicators to comprehensively evaluate acupuncture effects (symptoms, functional status, quality of life, laboratory parameters, and related aspects). Additionally, given the variety of acupuncture therapies available, it is essential to investigate which type provides greater benefits to patients.

## 5. Conclusion

Based on the outcomes analyzed in this study, acupuncture therapy demonstrated superior efficacy to sham/placebo acupuncture in PGD across multiple cancer types. Current evidence suggests acupuncture is well-tolerated in this context. The therapeutic effect may be influenced by variations in acupuncture modalities and sham/placebo acupuncture protocols. Acupuncture appears to be safe, but adverse events were often underreported. Consequently, further high-quality research with rigorous safety surveillance is needed to establish its overall safety profile.

## Acknowledgments

Our most excellent acknowledgments is to the authors who provided extensive data for our meta-analysis, as well as all of our colleagues in this investigation.

## Author contributions

**Conceptualization:** Weiai Liu.

**Data curation:** Mohao Zhu, Mengxue Liu, Weiai Liu.

**Formal analysis:** Lin Chen, Weiai Liu.

**Investigation:** Mohao Zhu, Lin Chen.

**Methodology:** Mohao Zhu, Lin Chen, Weiai Liu.

**Project administration:** Weiai Liu.

**Resources:** Weiai Liu.

**Software:** Mohao Zhu, Mengxue Liu.

**Supervision:** Weiai Liu.

**Visualization:** Xiaolong Peng, Ying Liu.

**Writing – original draft:** Mohao Zhu.

**Writing – review & editing:** Mohao Zhu, Lin Chen.

## Supplementary Material


