# Supplementary material for: A systematic review and meta-analysis of acupuncture versus sham/placebo acupuncture for postoperative gastrointestinal dysfunction in cancer patients: Evidence from randomized controlled trials
Source: Medicine (Baltimore). 2026 Jan 23;105(4):e47305. doi: 10.1097/MD.0000000000047305 (PMC12851784; doi:10.1097/MD.0000000000047305)
Supplement: Supplementary file 1 [file medi-105-e47305-s001.pdf]

A systematic review and meta-analysis of acupuncture versus sham/placebo acupuncture for postoperative gastrointestinal dysfunction in cancer patients: evidence from randomized controlled trials

Mohao Zhu[1,2], Lin Chen[3],Xiaolong Peng[1,2],Mengxue Liu[1,2],Ying Liu[1,2],And Weiai Liu[1,2]

1 The Second Affiliated Hospital of Hunan University of Chinese Medicine, Changsha, Hunan, China,

2 The Second Clinical College of Hunan University of Chinese Medicine, Changsha, Hunan, China

3 The Second Xiangya Hospital of Central South University ,Changsha, Hunan, China

*Correspondence:**

Corresponding:Weiai Liu

doctorlwa@163.com

(1) The search strategy of PubMed

NO.	Search Items
**#1**	Neoplasms[MeSH Terms]
**#2**	(((((((((((Tumor[Title/Abstract]) OR (Neoplasm[Title/Abstract])) OR (Tumors[Title/Abstract])) OR (Neoplasia[Title/Abstract])) OR (Neoplasias[Title/Abstract])) OR (Cancer[Title/Abstract])) OR (Cancers[Title/Abstract])) OR (Malignant Neoplasm[Title/Abstract])) OR (Malignancy[Title/Abstract])) OR (Malignancies[Title/Abstract])) OR (Malignant Neoplasms[Title/Abstract])) OR (neoplasm malignant[Title/Abstract])) OR (neoplasms malignant[Title/Abstract])
**#3**	**#1 OR #2**
**#4**	Postoperative Complications[MeSH Terms]
**#5**	(((((Postoperative[Title/Abstract])) OR (Post-operative[Title/Abstract])) OR (Post-surgical[Title/Abstract])))
**#6**	**#4 OR #5**
**#7**	Gastrointestinal Diseases[MeSH Terms]
**#8**	(((((Gastrointestinal Disease[Title/Abstract])) OR (Gastrointestinal Disorders[Title/Abstract])) OR (Gastrointestinal Disorder[Title/Abstract])) OR (Gastrointestinal Dysfunction[Title/Abstract]))
**#9**	**#7 OR #8**
**#10**	Acupuncture Therapy[MeSH Terms]
**#11**	((Acupuncture Treatment[Title/Abstract]) OR (Pharmacoacupuncture Treatment[Title/Abstract])) OR (Pharmacoacupuncture Therapy [Title/Abstract])

**#12**	**#10 OR #11**
**#13**	((((((((((((Acupuncture[Title/Abstract]) OR (Electroacupuncture[Title/Abstract])) OR (Electro-acupuncture[Title/Abstract])) OR (Electric acupuncture [Title/Abstract])) OR (Acupoint[Title/Abstract])) OR (Ear Needle[Title/Abstract])) OR (Auricular Needle[Title/Abstract])) OR (Wrist Ankle Needle[Title/Abstract])) OR (Acupoint Embedding[Title/Abstract])) OR (Acupoint Injection[Title/Abstract])) OR (Acupoint Sticking[Title/Abstract])) OR (Needle Knife[Title/Abstract])
**#14**	**#12 OR #13**
**#15**	Randomized Controlled Trial[MeSH Terms]
**#16**	((((Clinical Study[Title/Abstract])) OR (Clinical Trial[Title/Abstract])) OR (Controlled Clinical Trial[Title/Abstract]))
**#17**	placebo [Title/Abstract]
**#18**	**#15 OR #16 OR #17**
**#19**	**#3 AND #6 AND #9 AND #14 AND #18**

(2) The search strategy of Embase

Embase <1988 to 2024 Week 35>

NO.	Search Items
#1	(Neoplasms or Tumor or Neoplasm or Tumors or Neoplasia or Neoplasias or Cancer or Cancers or Malignant Neoplasm or Malignancy or Malignancies or Malignant Neoplasms or neoplasm malignant or neoplasms malignant)
#2	(Postoperative Complications or Postoperative or Post-operative or Post-surgical)
#3	(Gastrointestinal Diseases or Gastrointestinal Disease or Gastrointestinal Disorders or Gastrointestinal Disorder or Gastrointestinal Dysfunction)
#4	(Acupuncture Therapy or Acupuncture Treatment or Pharmacoacupuncture Treatment or Pharmacoacupuncture Therapy or Acupuncture or Electroacupuncture or Electro-acupuncture or Electric acupuncture or Acupoint or Ear Needle or Auricular Needle or Wrist Ankle Needle or Acupoint Embedding or Acupoint Injection or Acupoint Sticking or Needle Knife)
#5	(Randomized Controlled Trial or Clinical Study or Clinical Trial or Controlled Clinical Trial or placebo)
#6	1 and 2 and 3 and 4 and 5 16

(3) The search strategy of Coherent

NO.	Search Items

**#1**	MeSH descriptor: [Neoplasms] explode all trees
**#2**	(Tumor):ti,ab,kw OR (Neoplasm):ti.ab,kw OR (Neoplasia):ti,ab,kw OR (Cancer):ti,ab,kw OR (Malignant Neoplasm):it,ab,kw
**#3**	(Malignancy):ti,ab,kw OR (Malignancies):ti.ab,kw OR (Malignant Neoplasms):ti.ab.kw OR (neoplasm maionant):ti.ab.kw OR (neop lasms malionant):ti,ab,kw
**#4**	**#1 or #2 or #3**
**#5**	MeSH descriptor: [Postoperative Complications] explode all trees
**#6**	(Postoperative Complications):ti,ab,kw OR (Postoperative):ti,ab,kw OR (Post-operative):ti,ab,kw OR (Post-surgical):ti,ab,kw
**#7**	**#5 or #6**
**#8**	MeSH descriptor: [Gastrointestinal Diseases] explode all trees
**#9**	(Gastrointestinal Disease):ti,ab,kw OR (Gastrointestinal Disorder);ti,ab,k w OR (Gastrointestinal Dysfunction):ti.ab.kw
**#10**	**#8 or #9**
**#11**	MeSH descriptor: [Acupuncture Therapy] explode all trees
**#12**	(Acupuncture Therapy):ti,ab,kw OR (Acupuncture Treatment):ti,ab,kw O R (Pharmacoacupuncture Treatment):ti,ab.kw OR (Pharmacoacupuncture Therapy):ti,ab,kw
**#13**	**#11 or #12**
**#14**	MeSH descriptor: [Acupuncture] explode all trees
**#15**	(Acupuncturel):ti,ab,kw OR (Electroacupuncture):ti,ab,kw OR (Electro-acupuncture):ti,ab,kw OR (Electric acupuncture):ti,ab,kw OR (Acupoint):ti,ab,kw
**#16**	(Ear Needle):ti,ab,kw OR (Auricular Needle):ti,ab,kw OR (wrist Ankle Needlel):ti,ab,kw OR (Acupoint Embedding):ti,ab,kw OR (Acupoint injection):ti, ab,kw
**#17**	(Acupoint Sticking):ti,ab,kw OR (Needle Knife):ti,ab,kw
**#18**	**#13 or#14 or#15 or#16 or#17**
**#19**	MeSH descriptor: [Randomized Controlled Triall explode all trees
**#20**	(Randomized Controled Trnial):ti,ab,kw OR (Clinical tudy):ti,ab,kw OR (Clinical Trial):ti,ab,kw OR (controled clinical Trial):ti,ab,kw OR (placebo):ti,ab,kw
**#21**	**#19 or #20**
**#23**	**#4 and #7 and #10 and #18 and #21**

(4) The search strategy of WOS

**NO.**	**Search Items**
**#1**	((((((((((((TS=(Tumor)) OR TS=(Neoplasm)) OR TS=(Tumors)) OR

	TS=(Neoplasia)) OR TS=(Neoplasias)) OR TS=(Cancer)) OR TS=(Cancers)) OR TS=(Malignant Neoplasm)) OR TS=(Malignancy)) OR TS=(Malignancies)) OR TS=(Malignant Neoplasms)) OR TS=(neoplasm malignant)) OR TS=(neoplasms malignant)
**#2**	((TS=(Postoperative)) OR TS=(Post-operative)) OR TS=(Post-surgical) OR TS=(Postoperative Complications)
**#3**	(((TS=(Gastrointestinal Disease)) OR TS=(Gastrointestinal Disorders)) OR TS=(Gastrointestinal Disorder)) OR TS=(Gastrointestinal Dysfunction) OR TS=(Gastrointestinal Diseases)
**#4**	((((((((((((TS=(Acupuncture)) OR TS=(Electroacupuncture)) OR TS=(Electro-acupuncture)) OR TS=(Electric acupuncture)) OR TS=(Acupoint)) OR TS=(Ear Needle)) OR TS=(Auricular Needle)) OR TS=(Wrist Ankle Needle)) OR TS=(Acupoint Embedding)) OR TS=(Acupoint Injection)) OR TS=(Acupoint Sticking)) OR TS=(Needle Knife)) OR TS=(Acupuncture Treatment) OR TS=(Acupuncture Therapy) OR TS=(Pharmacoacupuncture Treatment) OR TS=(Pharmacoacupuncture Therapy)
**#5**	((TS=(Clinical Study)) OR TS=(Clinical Trial)) OR TS=(Controlled Clinical Trial) OR TS=(Randomized Controlled Trial)
**#6**	**#1 and #2 and #3 and #4 and #5**

(5) The search strategy of CNKI

In Chinese

篇关摘：“肿瘤 + 瘤子 +瘤 +虫瘿 +癌”and “术后” and“胃肠功能 + 胃肠道功能” and “针灸 + 温针灸 + 电针 + 头针 +经皮穴位电刺激 + 腕踝针 +耳针 +揿针” and “随机对照试验 + 随机对照实验 + 随机对照研究”

(6) The search strategy of VIP:

In Chinese

(((((((任意字段=肿瘤 OR 任意字段=瘤子) OR 任意字段=瘤) OR 任意字段=虫瘿) OR 任意字段=癌) AND 任意字段=术后) AND (任意字段=胃肠功能 OR 任意字段=胃肠道功能)) AND (((((((任意字段=针灸 OR 任意字段=温针灸) OR 任意字段=电针) OR 任意字段=头针) OR 任意字段=经皮穴位电刺激) OR 任意字段=腕踝针) OR 任意字段=耳针) OR 任意字段=揿针) AND(随机对照试验 or 随机对照实验 or 随机对照研究))

(7) The search strategy of WanFang

In Chinese

全部:(肿瘤 or 瘤子 or 瘤 or 虫瘿 or 癌) and 全部:(术后) and 全部:(胃肠功能 or 胃肠道功能) and 全部:(针灸 or 温针灸 or 电针 or 头针 or 经皮穴位电刺激 or 腕踝针 or 耳针 or 揿针) and 全部:(随机对照试验 or 随机对照实验 or 随机对照研究)

(8) The search strategy of CBM

In Chinese

("随机对照试验"[全部字段:智能] OR "随机对照研究"[全部字段:智能] OR "随机对照实验"[全部字段:智能]) AND ("针灸"[全部字段:智能] OR "针灸疗法"[全部字段:智能] OR "艾灸"[全部字段:智能] OR "温针灸"[全部字段:智能] OR "电针"[全部字段:智能] OR "头针"[全部字段:智能] OR "经皮穴位电刺激"[全部字段:智能] OR "腕踝针"[全部字段:智能] OR "耳针"[全部字段:智能] OR "揿针"[全部字段:智能] OR "穴位"[全部字段:智能] OR "艾灸"[全部字段:智能] OR "针刺"[全部字段:智能] OR "穴位按压"[全部字段:智能]) AND ("胃肠道功能"[全部字段:智能] OR "胃肠功能"[全部字段:智能] OR "胃肠"[全部字段:智能]) AND ("癌症"[全部字段:智能] OR "肿瘤"[全部字段:智能] OR "癌"[全部字段:智能] OR "瘤"[全部字段:智能]) AND ("术后"[全部字段:智能] OR "手术"[全部字段:智能])

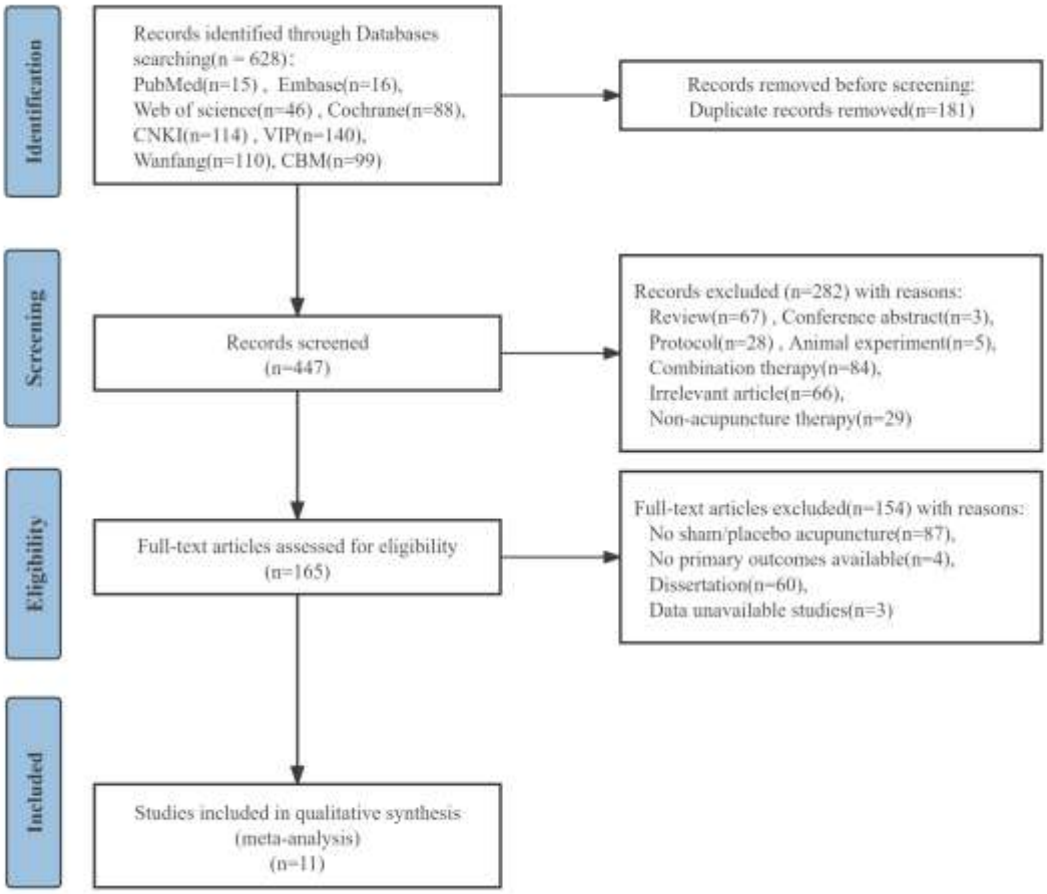
Identification
Records identified through Databases searching(n = 628):
PubMed(n=15) , Embase(n=16),
Web of science(n=46) , Cochrane(n=88),
CNKI(n=114) , VIP(n=140),
Wanfang(n=110), CBM(n=99)
Records removed before screening:
Duplicate records removed(n=181)
Screening
Records screened
(n=447)
Records excluded (n=282) with reasons:
Review(n=67) , Conference abstract(n=3),
Protocol(n=28) , Animal experiment(n=5),
Combination therapy(n=84),
Irrelevant article(n=66),
Non-acupuncture therapy(n=29)
Eligibility
Full-text articles assessed for eligibility
(n=165)
Full-text articles excluded(n=154) with reasons:
No sham/placebo acupuncture(n=87),
No primary outcomes available(n=4),
Dissertation(n=60),
Data unavailable studies(n=3)
Included
Studies included in qualitative synthesis
(meta-analysis)
(n=11)

Author Statement

We declare that this manuscript is original, has not been published before and is not currently being considered for publication elsewhere.

We confirm that the manuscript has been read and approved by all named authors and that there are no other persons who satisfied the criteria for authorship but are not listed. We further confirm that the order of authors listed in the manuscript has beenapproved by all of us.

We understand that the Corresponding Author is the sole contact for the Editorial process. She is responsible for communicating with the other authors about progress, submissions of revisions and final approval of proofs.

All authors as follows:

Author 1 (Mohao Zhu): Writing – review & editing, Writing – original draft, Software, Methodology, Investigation, Data curation.

Author 2 (Lin Chen): Writin – review & editing, Methodology, Investigation, Formal analysis.

Author 3 (Xiaolong Peng): Visualization.

Author 4 (Mengxue Liu): Software, Data curation.

Author 5 (Ying Liu): Visualization.

Author 6 (Weiai Liu, Corresponding Author):Supervision, Resources, Project administration, Methodology, Formal analysis, Data curation, Conceptualization.
